# Low-dose apatinib in combination with chemotherapy for hormone receptor-positive, HER2-negative breast cancer with pulmonary lymphangitic carcinomatosis: A case report

**DOI:** 10.1097/MD.0000000000040345

**Published:** 2024-11-08

**Authors:** Liping Chen, Sha Feng, Xuelian Chen, Caiwen Du

**Affiliations:** a Department of Medical Oncology, National Cancer Center/National Clinical Research Center for Cancer/Cancer Hospital & Shenzhen Hospital, Chinese Academy of Medical Sciences and Peking Union Medical College, Shenzhen, Guangdong, China; b Department of Pathology, National Cancer Center/National Clinical Research Center for Cancer/Cancer Hospital & Shenzhen Hospital, Chinese Academy of Medical Sciences and Peking Union Medical College, Shenzhen, Guangdong, China.

**Keywords:** apatinib, case report, HR-positive HER2-negative metastatic breast cancer, pulmonary lymphangitic carcinomatosis, visceral crisis

## Abstract

**Rationale:**

Hormone receptor-positive, HER2-negative advanced breast cancer complicated by pulmonary lymphangitic carcinomatosis (PLC) poses significant therapeutic challenges due to the lack of standardized treatment protocols. Despite various therapeutic interventions and supportive care, prognosis remains dismal.

**Patient concerns:**

Herein, a 48-year-old Chinese woman presented with a persistent cough, unresponsive to anti-infective treatment for 1 month. A computed tomography (CT) scan revealed lymphatic vessel infiltration and a diffuse nodular pattern, suggestive of PLC.

**Diagnoses:**

Hormone receptor-positive, HER2-negative advanced breast cancer complicated by PLC.

**Interventions:**

The patient was treated with a regimen comprising low-dose apatinib, capecitabine, and albumin-bound paclitaxel.

**Outcomes:**

The patient achieved a partial response, with a progression-free survival exceeding beyond ten months. Symptoms of dyspnea and dry cough significantly improved, alongside a notable reduction in lymphangitic carcinomatosis.

**Lessons:**

This case highlights the potential antitumor activity of apatinib in breast cancer patients with presenting with PLC. While further studies are necessary, this therapeutic approach could represent a viable option for managing breast cancer in the context of a visceral crisis. The case also emphasizes the importance of individualized treatment strategies and further research to substantiate these promising findings.

## 1. Introduction

Breast cancer is the most common malignancy among women and remains the leading cause of cancer-related mortality in females worldwide.^[1]^ Approximately 70% of human breast tumors express hormone receptors (HRs), including the estrogen receptor (ER) and/or progesterone receptor (PR).^[2]^ The advent of cyclin-dependent kinase 4/6 inhibitors (CDK4/6i), such as palbociclib, ribociclib, abemaciclib, and dalpiciclib, has significantly extended survival in patients with HR-positive, HER2-negative metastatic breast cancer (MBC).^[3–6]^ Current international guidelines recommend the use of CDK4/6i in combination with endocrine therapy for HR-positive MBC, except in cases of visceral crisis.^[7–9]^ The 2022 Chinese Society of Clinical Oncology (CSCO) Breast Cancer Guidelines further specify that chemotherapy is the preferred treatment in HR-positive patients experiencing visceral crises.^[10]^

Although chemotherapy is typically recommended for advanced breast cancer characterized by rapid progression or severe symptoms, including life-threatening visceral involvement, the management of MBC with visceral metastasis remains challenging. Approximately 10% to 15% of HR-positive, HER2-negative breast cancer patients experience visceral crises,^[11]^ which may present with diffuse liver metastases, meningeal involvement, bone marrow infiltration, or pulmonary lymphangitic carcinomatosis (PLC). PLC is a condition where cancer cells spread through the lymphatic vessels of the lungs, leading to infiltration and blockages that cause respiratory symptoms such as cough and shortness of breath. It is often considered a sign of terminal-stage malignancy.^[12]^ In a retrospective study of 27 patients with pathologically confirmed PLC, the median survival time postdiagnosis was only 5.7 months.^[13]^

Vascular endothelial growth factor (VEGF), a key angiogenic mediator, is secreted by many solid tumors and has been shown to play a critical role in the progression of PLC.^[14]^ Apatinib, a tyrosine kinase inhibitor, inhibits vascular endothelial cell proliferation and migration, reducing tumor microvascular density by selectively targeting the vascular endothelial growth factor receptor-2 (VEGFR-2).^[15]^ Here, we report a case of a female patient diagnosed with HR-positive MBC with lymphangitic carcinomatosis, who was treated with low-dose apatinib, capecitabine, and albumin-bound paclitaxel as first-line systemic therapy. To our knowledge, this is the first documented case of apatinib combined with chemotherapy as a systemic treatment for HR-positive MBC with PLC.

## 2. Case report

In June 2019, a 48-year-old Chinese woman underwent a mastectomy and homolateral axillary dissection for invasive ductal carcinoma of the right breast. Pathological evaluation revealed grade II invasive nonspecific breast carcinoma, with the largest tumor measuring 33 mm in diameter. Of the 37 excised right axillary lymph nodes, 10 showed metastatic involvement. No distant metastases were identified. Postoperative staging was classified as pT2N3M0, stage IIIC. Immunohistochemistry indicated 90% ER positivity and 80% PR positivity, with no amplification of the HER2 gene, confirming the molecular subtype as HR-positive, HER2-negative breast cancer. The proliferation index was elevated (Ki-67 = 30%). The patient did not undergo genetic testing for germline mutations, such as BRCA1 or BRCA2. There was no significant family history of cancer or other comorbidities. The patient declined adjuvant chemotherapy, radiotherapy, and endocrine therapy.

**Figure 1.**
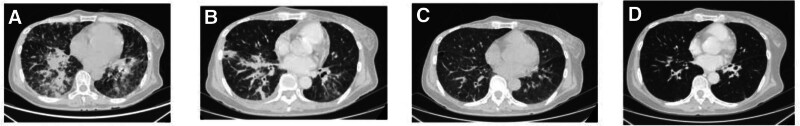
Sequential chest computed tomography (CT) scans delineate the changes of pulmonary lymphangitic carcinomatosis (PLC). (A) Prior to the initiation of treatment. (B) Subsequent to 2 cycles of apatinib combined with chemotherapy. (C) Following 4 cycles of apatinib combined with chemotherapy. (D) After the completion of 8 cycles of apatinib and chemotherapy.

**Figure 2.**
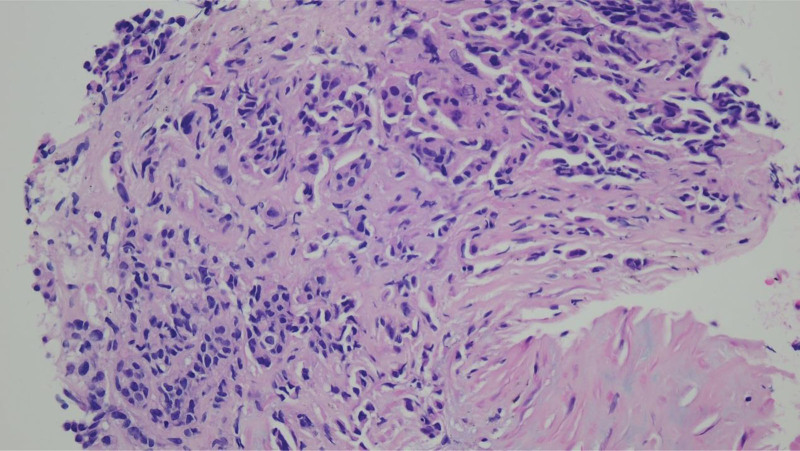
Pathological results of supraclavicular lymph node biopsy.

In May 2023, the patient presented to an external hospital with a persistent cough that was unresponsive to anti-infective treatments. One month later, a positron emission tomography/computed tomography (PET/CT) scan at another hospital revealed multiple bilateral pulmonary lesions, as well as extensive bone and lymph node metastases. The patient was subsequently admitted to our hospital due to worsening dyspnea and a dry cough. A baseline CT scan demonstrated infiltration and diffuse nodular distribution along the lymphatic vessels, indicative of PLC (Fig. 1A). Multiple lymph node and bone metastases were also confirmed. Brain MRI identified numerous cerebral metastases. In June 2023, a supraclavicular lymph node biopsy confirmed MBC (Fig. 2). Immunohistochemical analysis showed ER (90%+), PR (60%+), HER2 (1+), and Ki-67 (60%), consistent with HR-positive, HER2-negative breast cancer. Given the progression of the disease, the patient was diagnosed with a visceral crisis. For brain metastases, the patient refused radiotherapy.

On June 30, 2023, the patient began first-line treatment with capecitabine (1.5 g, orally, twice daily, days 1 to 14 of a 3-week cycle) and albumin-bound paclitaxel (130 mg/m², intravenous, on days 1 and 8 of a 3-week cycle). However, after 1 cycle of chemotherapy, the patient’s dry cough and dyspnea worsened. Consequently, low-dose apatinib was added to the treatment regimen on July 7, 2023. Remarkable clinical improvement in breathlessness and cough was observed 3 days posttherapy. A follow-up chest CT after 2 cycles of the combination therapy revealed significant reduction of the pulmonary lesions (Fig. 1B). After 4 and 8 cycles, the pulmonary lesions showed substantial shrinkage (Fig. 1C and D). The overall treatment response was classified as a partial response.

**Figure 3.**
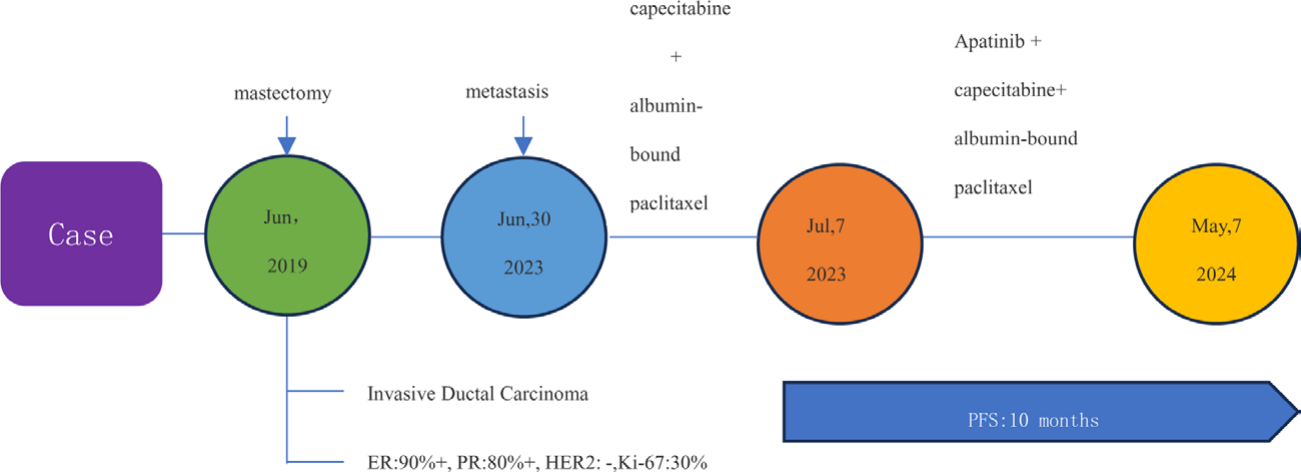
The timeline of the patient^’^s treatment strategy.

The patient tolerated the treatment well, with only grade 1 hand-foot syndrome observed. No grade 3 or 4 adverse events were reported throughout the treatment period. As of the cutoff date, May 1, 2024, the patient has experienced over ten months of clinical benefit from low-dose apatinib. A timeline summarizing the patient’s treatment and response is presented in Figure 3.

## 3. Discussion

We report a case in which the combination of apatinib and chemotherapy resulted in a significant improvement in PLC. PLC is characterized by the spread of malignant cells through the pulmonary vasculature and lymphatics from a primary tumor, occurring in approximately 17.3% of MBC cases.^[16]^ As PLC is associated with end-stage malignancy and a poor prognosis, early response to treatment is critical. However, PLC often responds suboptimal to standard chemotherapy, and no established treatment protocols currently exist for its management.

Theories regarding the dissemination of metastatic tumors into the lymphatic system are varied. One hypothesis suggests that tumor cells initially spread via the bloodstream, causing occlusive endarteritis, and subsequently infiltrate the vascular endothelium to access the lymphatic channels. This process leads to local obstruction and fluid accumulation, as tumor cells become trapped within the lymphatic vessels.^[17]^ On a molecular level, VEGF, secreted by tumor cells, is thought to bind to VEGF receptors on endothelial cells within the lymphatic vessels, promoting lymph angiogenesis and accelerating the spread of tumor cells through the lymphatics.^[18]^

Apatinib acts by selectively targeting VEGFR-2, competing for adenosine triphosphate binding sites, thereby inhibiting tumor angiogenesis. Although apatinib is currently approved for advanced gastric cancer.^[19,20]^ Its efficacy has also been observed in MBC. Clinical studies have demonstrated that apatinib can be effective in MBC patients who are unresponsive to other therapies, with manageable toxicity profiles.^[21]^ In a multicenter phase II trial involving non-triple-negative MBC, apatinib monotherapy at 500 mg/day achieved an objective response rate of 16.7% and a median overall survival of 10.3 months.^[22]^ However, its effectiveness in treating PLC remains unclear.

Increasing evidence suggests that the VEGF-A/VEGFR-2 signaling pathway plays a crucial role in both lymph angiogenesis and lymphatic metastasis. In vitro studies have demonstrated that VEGF-A promotes the proliferation of lymphatic endothelial cells, while blocking VEGFR-2 can inhibit VEGF-A-induced proliferation, indicating that VEGF-A may facilitate lymph angiogenesis through VEGFR-2.^[23]^ In animal models, particularly mice with VEGF-A overexpression in skin cancer, VEGF-A has been shown to enhance VEGFR-2 activity in tumor-associated lymphatics, thereby promoting metastasis to lymph nodes.^[24]^ These findings suggest that VEGF-A may be involved not only in angiogenesis but also in lymph angiogenesis via VEGFR-2, contributing to tumor growth and metastasis.

## 4. Conclusion

In conclusion, our case demonstrates that the addition of apatinib to chemotherapy can lead to significant long-term improvement in PLC. Apatinib may offer a viable therapeutic option in emergency clinical situations, particularly in cases of visceral crisis. However, further clinical studies are essential to validate these findings and establish the broader applicability of this treatment approach.

## Author contributions

**Resources:** Caiwen Du, Sha Feng.

**Writing – original draft:** Liping Chen.

**Writing – review & editing:** Liping Chen, Xuelian Chen, Caiwen Du.
